# HO-1 and Heme: G-Quadruplex Interaction Choreograph DNA Damage Responses and Cancer Growth

**DOI:** 10.3390/cells10071801

**Published:** 2021-07-16

**Authors:** Giacomo Canesin, Anindhita Meena Muralidharan, Kenneth D. Swanson, Barbara Wegiel

**Affiliations:** 1 Department of Surgery, Division of Surgical Oncology, Cancer Research Institute, Beth Israel Deaconess Medical Center, Harvard Medical School, Boston, MA 02214, USA; giacomo.canesin@gmail.com (G.C.); a.muralidharan@dkfz-heidelberg.de (A.M.M.); 2 Department of Neurology, Beth Israel Deaconess Medical Center, Harvard Medical School, Boston, MA 02214, USA; kswanson@bidmc.harvard.edu

**Keywords:** G-quadruplex, heme, nuclear signaling, transcriptional control

## Abstract

Many anti-cancer therapeutics lead to the release of danger associated pattern molecules (DAMPs) as the result of killing large numbers of both normal and transformed cells as well as lysis of red blood cells (RBC) (hemolysis). Labile heme originating from hemolysis acts as a DAMP while its breakdown products exert varying immunomodulatory effects. Labile heme is scavenged by hemopexin (Hx) and processed by heme oxygenase-1 (HO-1, *Hmox1*), resulting in its removal and the generation of biliverdin/bilirubin, carbon monoxide (CO) and iron. We recently demonstrated that labile heme accumulates in cancer cell nuclei in the tumor parenchyma of *Hx* knockout mice and contributes to the malignant phenotype of prostate cancer (PCa) cells and increased metastases. Additionally, this work identified Hx as a tumor suppressor gene. Direct interaction of heme with DNA G-quadruplexes (G4) leads to altered gene expression in cancer cells that regulate transcription, recombination and replication. Here, we provide new data supporting the nuclear role of HO-1 and heme in modulating DNA damage response, G4 stability and cancer growth. Finally, we discuss an alternative role of labile heme as a nuclear danger signal (NDS) that regulates gene expression and nuclear HO-1 regulated DNA damage responses stimulated by its interaction with G4.

## 1. Introduction

The evolution and maintenance of a permissive tumor immune microenvironment (TIME) is essential for cancer evolution. The dynamics of the interactions among tumor and resident immune and non-immune stroma cells is now appreciated to dictate clinical outcomes in response to a variety of treatments. A primary axis that influences the functions within the TIME and as well as response to treatment involves the production and response to DAMPs. We and others have found that labile heme functions as a DAMP through its interactions with toll like receptors (TLRs) and as a NDS via binding to regulatory DNA structures that control stress, survival and proliferation related gene sets. In addition, many genes associated with promoting transformed phenotypes are increasingly identified with immune modulatory functions that also affect the TIME and have also been shown to be regulated by heme.

**Figure 1:**
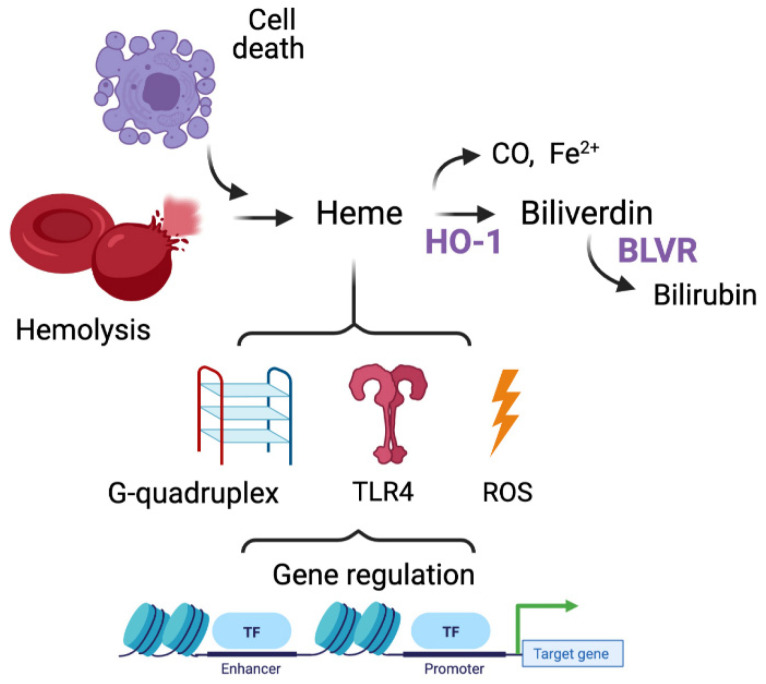
A model explaining the role of heme metabolism in the regulation of heme levels and cancer growth. Labile heme released from dying cells and/or erythrocytes (hemolysis) regulates gene expression via G4 DNA binding, ROS generation and TLR4 binding. BLVR- biliverdin reductase.

Labile heme released from dying RBC or necrotic cells is scavenged by Hx and delivered to macrophages (Mϕ) for its degradation by heme oxygenase-1 (HO-1, *Hmox1*) to immunomodulatory products: carbon monoxide (CO), iron and the bile pigments, biliverdin (BV)/bilirubin (BR) (Figure 1). *Hmox1* is a stress-induced gene, but it is also expressed basally in resident Mϕ [1,2,3,4] and regulates inflammatory responses during infection [5], tissue injury [5,6,7] and carcinogenesis [1,8]. We have recently shown that Mϕ either lacking *Hmox1* (*LysM-Cre:Hmox1^flfl^*) or exposed to heme exhibited a marked senescent phenotype, increased p16^INK4a^ expression and DNA damage [9,10]. Further, Mϕ lacking *Hmox1* are deficient in their ability to undergo maturation and polarization [2,11], to activate multiple inflammation-related signaling pathways [12,13] and to clear pathogens as well as to release cytokines [5].

Labile heme is a constituent of the cell debris or hemolyzed RBC and can drive inflammation as a DAMP via binding to toll-like receptor 4 (TLR4) and promote oxidative stress in the cell due to presence of reactive ferrous ions (Figure 1) [14]. Low levels of HO-1, its altered subcellular localization (nuclear isoform), or low levels of Hx are associated with increased labile heme levels within the TIME [15,16,17]. Repeated heme exposures may occur due to bleeding during cancer progression, trauma, or hemorrhage (i.e., surgical removal of the tumor). Recently, Panigrahy et al. has demonstrated that tumor cells killed by chemotherapy or targeted therapy (“tumor cell debris”) stimulate cancer growth in a model of tumor dormancy [18]. Our published data indicate that labile heme or lack of Hx in the tumor stroma clearly promote cancer progression [10]. This might be in part due to immune imbalance, but primarily due to altered gene expression in response to heme:G4 interaction within the promoter regions of key oncogenes including *c-MYC* [19] as well as heme-induced HO-1 expression.

Our recent findings emphasize the importance of labile heme in the direct regulation of gene expression via modulation of G4 stability [19] (Figure 1). G4 are well-defined secondary DNA or RNA structures resulting from Hoogstein hydrogen base pairings between consecutive guanine nucleic acids coordinated with a metal cation [20,21]. G4 structures are found throughout the genome and play regulatory roles during transcription [22], recombination [23,24] and replication [25,26,27]. As such, G4 structures act as key regulators of oncogenes and cancer-driving gene promoters including *KRas*, *c-MYC*, *bcl2*, *PDGF-A*, *Rb*, *VEGF-A*, *hTERT* [28,29] and telomeres [30,31,32,33,34,35]. G4 structures form transiently during S phase of the cell cycle when DNA is temporarily single stranded and are subsequently unwound by endogenous helicases. G4 regions are known to drive genomic instability [36] and application of G4 ligands (such as pyridostatin) in cell culture promotes DNA damage, which is marked by increase in γH2AX staining [30].

Heme has been shown to stabilize G4 in vitro contributing to π-π planar stacking interactions and metal coordination by the central Fe^2+^ atom of the porphyrin ring [37]. Recent studies further support this hypothesis and show that G4 DNA sequester free heme [38] to prevent from its accumulation and pro-oxidant activity.

Alteration of gene expression due to genetic mutation, deletion, amplification or abnormalities in chromatin/DNA structure is a hallmark of cancer [39]. Programs of gene regulation in normal cell are fundamental to coordinate synthesis of RNA and its subsequent translation. This is achieved by a multi-step and highly interconnected mechanisms involving transcriptional control, mRNA capping, splicing and editing and finally exporting the mRNA from the nucleus to the cytoplasm for translation [40]. In addition to the well-recognized changes in the DNA template, epigenetic modification such as histone acetylation or methylation impact chromatin structure and define transcriptional profiles in cancer cells [41]. The most studied epigenetic abnormalities in solid cancer are variation in DNA methylation, alterations in histone proteins structure through post-translational modifications and histone variants. Such changes in the epigenetic code are partially caused by metabolic reprogramming in cancer cells [42]. One of the examples of the alterations of gene expression checkpoint is amplification and transcriptional dysregulation of c-*MYC* oncogene, which is accompanied by an anabolic transcriptional response driving proliferation and metabolic adaptation [43]. Majority of transcriptional activity of the *c-MYC* promoter is controlled by formation of G4 in the proximal region [28,29]. We have demonstrated that heme intercalates into the G4 DNA structure in the *c-MYC* promoter and affects its stability and function [19].

In this short communication, we will discuss the role of nuclear HO-1 and heme in cancer, providing new evidence for their roles in DNA damage response, regulation of G4 dynamics and cancer growth. Specifically, we found that overexpression of truncated nuclear HO-1 promotes prostate cancer colony growth as efficiently as heme alone. It is well-appreciated that heme promotes nuclear HO-1, adding to the possible mechanisms of heme-induced anchorage independent growth. However, we did not see any measurable effects of a knockdown of HO-1 on colony growth in the absence or presence of heme. We demonstrated increased DNA damage marked by γH2AX foci in cells treated with heme and its colocalization with HO-1 staining in the nucleus. Indeed, HO-1 and γH2AX co-precipitated in cells treated with heme. We also demonstrated helicase activity associated with these HO-1:γH2AX complexes. These data suggest a mechanism via which HO-1/heme shape gene expression and downstream signaling during carcinogenesis.

## 2. Role of Heme Metabolism and Nuclear HO-1 in Gene Expression Control

Heme is both directly and indirectly involved in the control of transcription, DNA replication and various aspects of cellular metabolism. Heme is a co-factor and regulatory element of hemoproteins including mitochondrial cytochrome complexes and several nuclear transcription factors and nuclear enzymes such as Rev-Erbα, NPAS2, Bach1 and Drosha [44,45,46,47,48]. The presence of labile heme in the nucleus has been shown using intracellular heme sensors [49,50]. Further, multiple heme transporters have been identified [49,50]. A recently discovered nuclear heme receptor-transporter PGRMC2 has been described in adipose tissues [51] but has not been studied in cancer or other cell types. Our most recent data show that heme accumulates in the nucleus of cancer cells, with lower levels observed in non-transformed cells and drives early-response G4-regulated gene expression. Interestingly, in mice lacking Hx (*Hx^-/-^* mice) or treated with labile heme, orthotopic TRAMP C1 prostate tumors grew more rapidly and were more metastatic [19]. Moreover, analysis of 341 human prostate cancer specimens revealed a correlation between low stromal Hx or low G4 levels in cancer cells and poor prognosis. We also demonstrated that 60% of the heme-targeted genes displayed G-quadruplexes (G4)-rich promoters, supporting a possible direct regulation of gene expression through heme binding to these structures [19].

Labile nuclear heme levels are controlled in part by the activity of HO-1, whose expression is responsive to a variety of stimuli including ROS, endotoxins, heavy metals and hypoxia and by heme itself [17]. Nuclear HO-1 possesses a lower catalytic activity compared to cytoplasmic HO-1, which readily degrades heme [52,53,54]. While nuclear HO-1 has limited enzymatic activity in cancer cells, it has been described as a downstream mediator of the activating transcription factor-4 (ATF-4), controlling cancer cell death and metastasis through anoikis [52,53,54]. Of note, high expression of nuclear HO-1 has been shown to correlate with poor prognosis of prostate, lung and skin cancers and leukemia [53,55,56]. 

**Figure 2:**
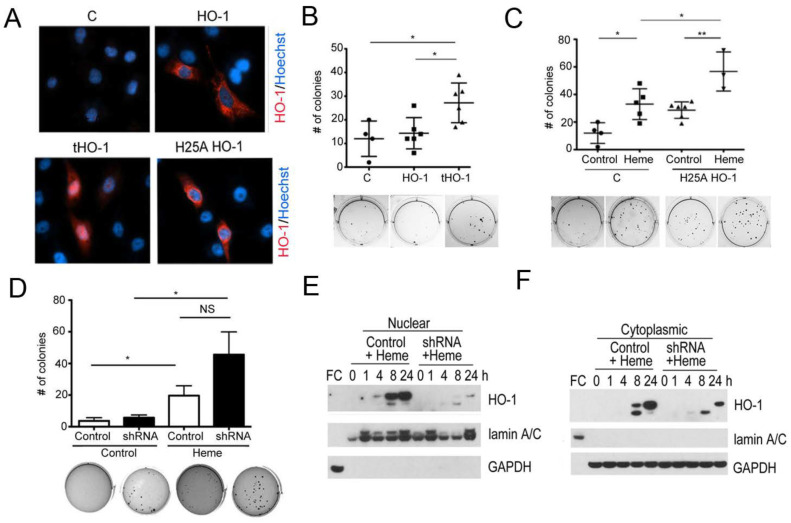
Nuclear HO-1 accelerates growth of PC3 cells. (**A**) Immunofluorescence analyses of PC3 cells overexpressing the Figure 1. the C-terminal 23 amino acid truncated (tHO-1), or the enzymatically inactive (H25A HO-1) HO-1. The C-terminal truncated tHO-1 localizes in the nucleus (bottom-left panel), while the full length (HO-1) and the enzymatically inactive H25A HO-1 localize in the cytoplasm of PC3 cells (top-right and bottom-right panel, respectively) compared to non-transfected cells (top-left panel). (**B,C**) Anchorage-independent growth in soft agar of PC3 cells overexpressing HO-1, tHO-1 or H25A HO-1 with or without heme treatment (50 μM) for 3 weeks. *n* = 3 independent experiments in triplicates. * *p* < 0.05, ** *p* < 0.01. (**D**–**F**). PC3 cells with knockdown of HO-1 (shRNA) or transfected with scramble shRNA (control) were treated with heme (50 μM) for 3 weeks and the number of colonies growing in soft agar was measured. Immunoblotting confirming the knockdown of HO-1 in the PC3 is shown in (**E**,**F**). The materials and methods part is stated in Supplementary Materials.

We have recently demonstrated the tumor-promoting role of labile heme in prostate cancer colony soft agar cultures, which was associated with an increase in nuclear HO-1 translocation [19]. To define the role of HO-1 in supporting prostate cancer colony growth in soft agar, we overexpressed a full-length HO-1 and truncated HO-1 (localized to the nucleus) in PC3 cells (Figure 2A). Full length HO-1 did not affect PC3 colony growth in soft agar, while nuclear truncated HO-1 (tHO-1) significantly increased the number of PC3 colonies (Figure 2A,B) suggesting that heme-induced nuclear HO-1 may mediate some of the effects of labile heme. Importantly, transfection with enzymatically inactive full-length HO-1 (H25A) promoted growth of PC3 colonies in soft agar (Figure 2C) and this synergized with heme to increase PC3 colony number (Figure 2C). Depletion of HO-1 by stable knockdown did not affect growth pattern of PC3 cells in the absence of heme, but resulted in a trend towards slightly higher number and larger colony growth upon heme treatment compared to control cell line; however, these differences were not significant (Figure 2D–F). These data support the role for heme-induced HO-1 in cancer progression and might be associated with early- and late-heme response gene expression [19].

## 3. Heme and G4 Interaction: Resolution by Helicases

Heme is a high-energy prosthetic group of hemoproteins such as transcription factors, gas carriers (i.e., hemoglobin), cytochromes and redox enzymes [57,58]. The mechanism by which nuclear heme induces mutations and/or alters gene expression beyond its ability to generate ROS due to the high reactivity of its ferrous ion in the Fenton reaction, is unclear [59]. Maintenance of steady state cellular heme levels is regulated by de novo heme synthesis by aminolevulinic acid (ALA) synthase (ALAS) and degradation of heme by the HO family of enzymes [60]. HO-1 also degrades heme from extracellular sources including aged erythrocytes and cellular debris [61]. Our previous work indicated the role of HO-1 and heme metabolites in DNA damage responses, which is important mechanisms controlling cancer development and progression [53,62]. The uptake of labile heme in a form of heme:Hx complexes is mediated by the myeloid cell-expressed receptor, CD91 (Low density lipoprotein receptor-related protein 1, LPR1) [63,64]. The liberated labile heme is transported from phagolysosomes to the cytoplasm via the heme-responsive gene-1 (HRG1) transporter [65], where it is degraded by HO-1. This is a well-established mechanism in phagocytotic macrophages, but not studied in the tumor microenvironment or cancer cells.

Nuclear heme interacts with G4 structures embedded in the promoter regions of key target genes, controlling their expression in cancer cells [19]. Previous work suggested that the porphyrin ring intercalates into the G4 and can affect G4 stability and function [37,66,67,68,69,70]. In vitro studies have shown that heme is able to coordinate with d(TTGAGG) oligonucleotides that form G4 structures. Further, G4 are known to sequester free heme but no molecular mechanisms of such interaction were provided [38]. We have recently demonstrated that heme binding to G4 mitigates the interaction between the *c-MYC* promoter and the G4-interacting proteins nucleolin, NM23-H2 and hnRNPK, resulting in increased *c-MYC* expression and facilitating tumor growth and metastasis [28]. This effect is transient, however, and limited by degradation of heme by HO-1, which is induced at 6–8 h upon heme treatment. This negative feedback mechanisms may be lacking in cancer cells due to low enzymatic activity of nuclear HO-1.

By employing RNAseq, we found that a wide variety of genes such as cell cycle regulators (growth factor signaling, cyclins and S-phase regulators) or epithelial to mesenchymal transition (EMT) inducers, including TGF-β and Wnt pathway proteins, were transiently up-regulated in response to heme. We were able to identify G4-rich promotor regions in sixty percent of these heme-responsive genes, which supports the hypothesis that heme directly intercalates into G4 within promoters of key genes to regulate tumor growth and metastatic spread.

**Figure 3:**
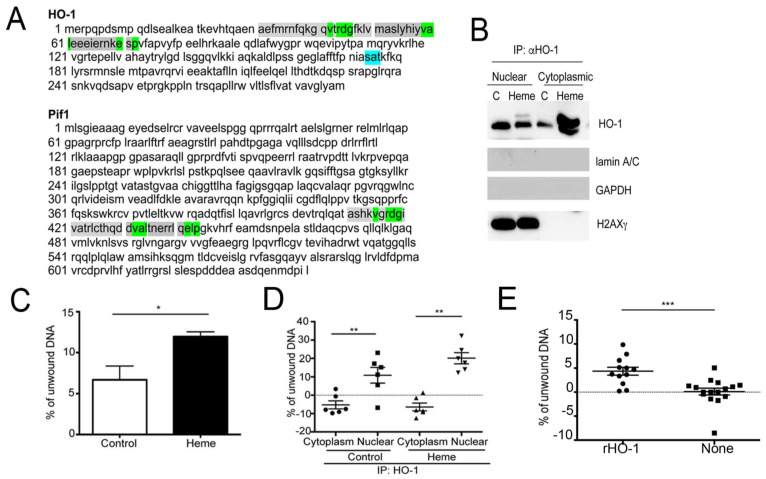
Helicase activity in heme treated cells- role of HO-1. (**A**) Similarities between the HO-1 and Pif1 sequences. The SAT motif of HO-1 is labeled. (**B**) Immunoprecpitation (IP) with antibody against HO-1 (α-HO-1, 1 µg) in the nuclear and cytoplasmic fractions of PC3 cells treated with heme (Heme, 50 µM) or control cells. (C) (24 h). (**C**) Helicase activity assays were performed in the lysates of PC3 cells treated with heme for 24 h as previously described [77]. * *p* < 0.05. Briefly, 1 µg sonicated genomic DNA was incubated with 1 mM ATP, 1× Sally Green, 10 g of protein lysates in the helicase buffer (200 mM Tris HCl pH 7.6, 25 mM MgCl2, 20 mM DTT, 125 mM KCl, 10% glycerol, 0.5 mg/mL BSA). Reactions were incubated at 37 °C for 30 min and fluorescence was measured at 492 and 530 nm. Percent of unwound substrate was calculated as described in [77]. (**D**) Helicase activity assays were performed in the immunoprecipitates of HO-1 in the nuclear and cytoplasmic fractions of PC3 cells treated with heme for 24 h. ** *p* < 0.01. *n* = 3 biological replicates. (**E**) Helicase activity of the recombinant HO-1 (Enzo Life Sciences) using 1 g of recombinant protein in the helicase assay as described above. *** *p* < 0.001.

G4 structures are resolved by members of the helicase superfamilies, including SF1, Pif1 or SF2, RecQ, Fanconi anemia group J protein (FANCJ), Bloom syndrome protein (BLM) and Werner syndrome protein (WRN) [71,72,73]. Pif1 helicase has been recently shown to be essential for G4 unwinding [74]. The absence or a non-functional Pif1 helicase (Pfh1) with an alcohol-alanine-alcohol (SAT) motif mutation is known to result in unresolved G4 structures, causing fork pausing and DNA damage as often seen in human tumors [75,76]. Nuclear heme may regulate a broad variety of genes by directly interacting with G4s in their promoters. Moreover, we propose that heme-driven expression of nuclear HO-1 may lead to unwinding of G4 structures due to increased helicase-like activity of HO-1 or HO-1-associated proteins in the nucleus. Interestingly, our analysis revealed that HO-1 has a highly conserved alcohol-alanine-alcohol (SAT) motif (Figure 3A). This SAT sequence (173–175 aa in human HO-1) is present in well-known DNA and RNA helicases and is similar to the SAT motif of Pif1 (Figure 3A). To investigate a possible role of nuclear HO-1 in resolving G4 complexes, we precipitated HO-1 from the nuclear and cytoplasmic fractions of PC3 cells using specific antibodies and found that a significant helicase activity was associated with HO-1 immunoprecipitates (Figure 3B–D). Significantly, heme treatment of the cells prior to lysis resulted in a 3-fold increase in helicase activity in these precipitates (Figure 3C). We also observed an increased helicase activity of HO-1 recombinant protein (Figure 3E), which may suggest a direct unwinding capacity of HO-1. These data also suggest that rather than acting primarily as helicase in the nucleus, HO-1 likely recruits more efficient helicases in vivo since the HO-1 immune precipitates exhibited 4–5-fold higher helicase activity compared to the HO-1 recombinant protein.

The above data indicate that nuclear HO-1 is an important effector protein in response to heme and may modulate G4 function by supporting temporary or permanent G4 unwinding. Our results are also in agreement with recent data showing that nuclear HO-1 co-localizes with G-quadruplexes and, similar to G4-unwinding helicases, it might be associated with lower G4 staining [78].

## 4. Heme Metabolism and Control of DNA Damage-Associated with G-Quadruplexes

DNA is prone to damage by UV light, radiation and various chemicals that promote generation of ROS. Double-stranded breaks (DSB) and single stranded breaks (SSB) are recognized by the ATM and ATR kinases resulting in activation of homologous recombination (HR) and non-homologous end joining (NHEJ). We have previously reported that H2AX phosphorylation (γH2AX), a marker for ongoing DNA damage or repair, was significantly increased in cells isolated from *Hmox1*^−/−^ mice [79]. The elevated levels of γH2AX were not only seen under basal conditions but were also augmented in these mice upon treatment with doxorubicin [79]. Knockdown of *Hmox1* gene in HEK cells resulted in the inhibition of ATM phosphorylation and delayed Brca1 activation in response to DNA damage, suggesting decreased ability to repair DNA breaks in the cells lacking HO-1. However, no significant change was observed in p53 phosphorylation. Similar effects were observed upon irradiation of HEK cells with knockdown of HO-1. This indicates that HO-1 may have a significant role in regulating apoptosis, cell cycle progression as well as key components in DNA damage repair pathway [79].

Heme generates ROS as a result of the Fenton reaction that can cause oxidative damage and DNA breaks. Induction of HO-1 and generation of CO appear to act as limiting factors in the DNA damage response signaling. We demonstrated that CO blocks chronic γH2AX foci formation in irradiated mice. Indeed, we showed a strong induction of phosphorylated p53, Brca1, or ATM and early induction of γH2AX in the bone marrow indicating early resolution of DNA damage and longer survival rate of CO-treated mice [79]. This strongly suggests that CO and HO-1 play a key role in eliciting a repair response after DNA damage.

**Figure 4:**
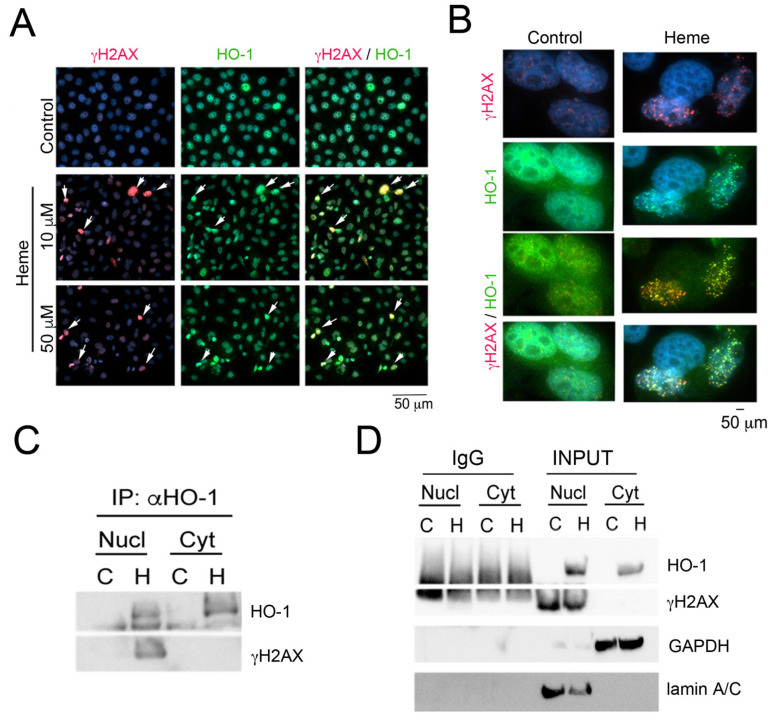
Heme induces DNA damage and accumulation of cells in the G2/M phases. (**A**,**B**) Immunofluorescent staining for HO-1 and γH2AX in PC3 cells treated with 50 μM heme for 24 h. *n* = 3 experiments (**A**—200×, **B**—630× magnifications). (**C**,**D**) Immunoprecipitation (IP) with antibody against HO-1 (α-HO-1) in the nuclear and cytoplasmic fractions of PC3 cells treated with heme (H) or control cells (**C**). Input proteins and IgG pull-downs are shown in (**D**). Results are representative of 3 independent experiments.

G4 are linked to genomic stability and thus, are implicated in the etiology of cancer and other pathologies [80]. We have shown that labile heme and HO-1 are key regulators of DNA damage: lack of HO-1 or accumulation of labile heme promotes chronic γH2AX foci accumulation [79]. Upon treatment with the G4 stabilizer, pyridostatin, genomic regions that contain G4 are marked by γH2AX positive foci [80]. These data are in agreement with our previous work showing that HO-1 knockout mice exhibited high levels of DNA damage [79]. Interestingly, HO-1 expression was detected 4 h after heme treatment and persisted for 24 h, while γH2AX phosphorylation was induced as early as 30 min after heme treatment (Figure 4A). We found both γH2AX phosphorylation and HO-1 proteins not only to overlap upon heme treatment (Figure 4A,B) but also to directly interact in the nucleus (Figure 4C,D). HO-1 may be involved in recruiting other proteins and helicases to the DNA damage foci or G4. Hence, heme:G4 or HO-1:heme:G4 complexes may attract co-activators of transcription or helicases capable of regulating gene expression via G4 stability. These questions will be addressed in future studies. Prior work demonstrated lower levels of G4 in normal hematopoietic cells deficient in HO-1 compared to wild type cells, which correlated with higher overall helicase levels (i.e., Brip1, Pif1) [78]. By using PLA technique in HEK293 cell line, it was demonstrated that HO-1 was localized in a proximity to G4 in the cytosol and to the much lesser extend in the nucleus and did not interact directly with G4 structures [78]. Here, we provided an explanation how HO-1 by interacting with γH2AX foci in cancer cells may impact G4 structures as G4 are marked by γH2AX positive foci [80]. Overall, these data suggest a strong link between the nuclear HO-1 function and response to DNA damage in cancer cells upon heme treatment.

### 4.1. Heme:G4 as Therapeutic Targets in Cancer

We have shown a strong association of low Hx levels and G4 in the tumor stroma with poor prognosis in a large cohort of prostate cancer biopsies. Moreover, we found higher levels of heme and lower levels of Hx in the plasma of cancer patients compared to healthy subjects. Mice lacking the peroxiredoxin-1 gene (*Prdx1^−/−^*) exhibit severe hemolysis and die prematurely experiencing high rates of malignancies, including sarcomas, lymphomas and carcinomas [81]. Heme was shown to induce hyperproliferation and aberrant atypical foci in the colon [82]. Similarly, extravascular RBC and hemoglobin have been shown to promote tumor growth by acting as DAMPs [83], through yet unknown mechanisms.

There is a high degree of relevance for hemolysis in many cancer types due to excessive angiogenesis and intra-tumoral hemorrhage. Repeated heme exposure may occur due to hemolysis, cell death (i.e., in response to chemotherapy, radiation) or administration of heme-arginate (to treat porphyrias). Increased vascular permeability, which is increased in the tumor due to leaky vessels, may lead to accumulation of high numbers of RBC and their poor re-cycling [84]. Therefore, it is possible that RBC poison the TIME with loads of labile heme and hemoglobin (labile heme concentration of 19–23 mM in each RBC). Hence, application of Hx to scavenge free heme might be a possibility as an adjuvant to therapy for cancer patients. Hx has been used in pre-clinical models of sickle cell anemia (SCD), which is characterized by hemolysis and increased load of labile heme, chronic inflammation and vaso-occlusive/painful crisis. SCD being a hemolytic disease causes lysis of erythrocytes which leads to the release of heme as a by-product from it pigment component hemoglobin [85,86]. The inflammation and vaso-occlusion seem to be mainly triggered by the excessive production of reactive oxygen species (ROS)- with heme as a major source. To counteract the toxic effects of heme, mammals are equipped with an extracellular scavenging system: Hx that binds labile heme and renders it inactive. In murine models, heme promotes inflammation by promoting the M1-pro-inflammatory phenotype of macrophages. Addition of Hx counteracts this heme-mediated effects on macrophage phenotype. Continued intraperitoneal injections with Hx into mice showed decrease in M1 macrophage marker expression such as MHC-II, Cd86 and IL-6 in a mouse model of SCD [87].

Increased microvascular stasis is counteracted by co-injection with haptoglobin (Hp) (a scavenger of hemoglobin), though combined injection of both Hx and Hp show same level of inhibition in stasis as they do individually [88]. Mechanistically, treatment with Hx has shown reduced activation of NF-κB, resulting in lower levels of other pro-inflammatory cytokines and higher expression of HO-1 [88]. Indeed, a dose dependent increase in HO-1 expression was observed in both liver and kidneys of mice injected with Hx or Hp. Furthermore, inhibition of vaso-occlusion was seen up to 48 h post injection with Hx, with a 5-fold increase in HO-1 at stasis induced site. Notably, the ameliorating effects of Hp and Hx were inhibited by the use of the HO-1 inhibitor protoporphyrin SnPP. Thus, Hp and Hx appear to inhibit stasis in SCD-mice by inducing HO-1 activity [88].

Based on the strong preclinical data, a plasma-derived form of Hx, CSL889, is being tested in a clinical trial as a treatment option for SCD to decrease the incidence of vaso-occlusive crisis and has been given Orphan drug status by both the European Commission and the Food and Drug Administration in the USA (ClinicalTrials.gov Identifier: NCT04285827).

### 4.2. Discussion and Future Directions

**Figure 5:**
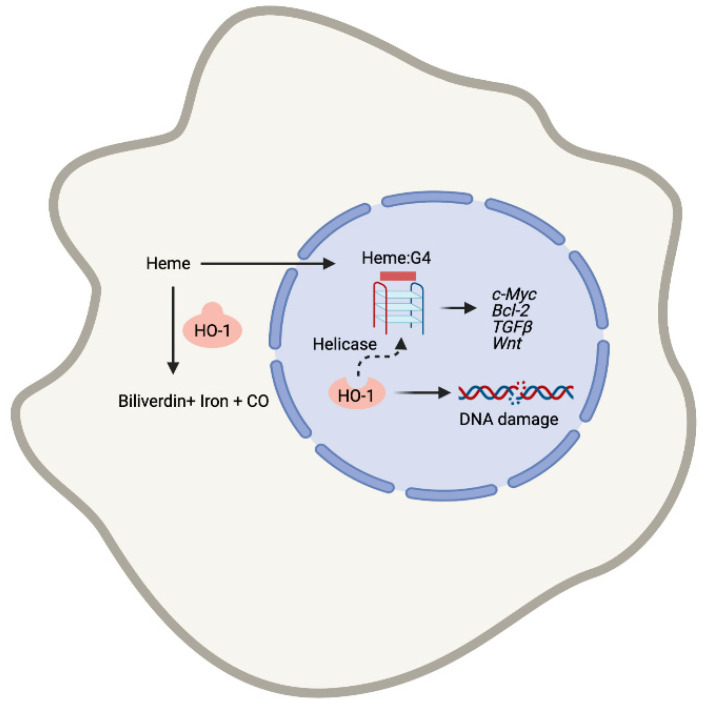
A scheme showing the role of nuclear heme in gene regulation of key genes (c*-MYC, BCl-2, TGFβ, Wnt*) as well as DNA damage signaling. An interaction of nuclear HO-1 with H2AX may be a site for recruiting factors (i.e., helicases) for repair and resolution of G4 structures, which are regulated by presence of nuclear heme.

Labile heme within the nucleus can play roles in gene regulation via direct interaction with G4 structures in DNA or RNA and DNA replication and repair. Importantly, not only is labile heme detected in the nucleus, but nuclear HO-1 is also readily detected in many types of cancer cells within tumors. Nuclear HO-1 has been shown to possess lower enzymatic activity but perform regulatory functions such as stabilization of Nrf2 [89]. Our data indicate that heme promotes the accumulation of nuclear HO-1 as previously reported [53] and further that nuclear tHO-1 increases prostate cancer colony growth in soft agar (Figure 5). Importantly, nuclear HO-1 not only co-localizes with γH2AX foci in response to heme, but our data strongly suggests that it promotes unwinding of G4 quadruplexes in the nucleus. These data indicate an increased genomic instability and shift of gene expression in response to heme-induced HO-1 in cancer cells. We propose that early heme-induced genes driven by heme:G4 interaction are a key drivers of carcinogenesis. Heme-induced HO-1 re-directs transcriptional activity in cancer cells in part by unwinding of heme:G4 complexes.

Moreover, reduction of HO-1 activity in the cytoplasm most likely allows for accumulation of higher levels of heme in the nucleus and higher colony growth. Therefore, enzymatically active HO-1 along with Hx might be a guardian against pathological cell growth by removal of cytoplasmic heme and/or by prevention of accumulation of nuclear heme.

## Supplementary Materials

References [89,90] are cited in the supplementary materials, https://www.mdpi.com/article/10.3390/cells10071801/s1.
