# Supplementary material for: HO-1 and Heme: G-Quadruplex Interaction Choreograph DNA Damage Responses and Cancer Growth
Source: Cells. 2021 Jul 16;10(7):1801. doi: 10.3390/cells10071801 (PMC8307061; doi:10.3390/cells10071801)
Supplement: Supplementary file 1 [file cells-10-01801-s001.zip › cells-1267828-supplementary.pdf]

1. Materials and Methods

1.1. Cell Culture and Cell Treatment

Prostate cancer PC3 cells were a gift from Dr. Steven Balk (BIDMC, Boston) and maintained in RPMI medium (Gibco, Life Technologies, Carlsbad, CA, USA) supplemented with 10% Fetal Bovine Serum (Atlanta Biologicals) as previously described [19]. PC3 LMP (Control) and PC3 mirHO-1 (shRNA against HO-1) were previously described [53]. For culture in the soft agar, 1×10^4 PC3 cells were suspended in 0.35% biotechnology grade agarose (Amresco, Solon, OH, United States) in RPMI supplemented with 10% FBS and plated before solidifying on a solid 0.5% agarose with RPMI supplanted with 10% FBS. Medium was replaced every third day. Colonies were maintained for 2–3 weeks in a 37 °C humidified incubator, after which they were stained with methylene blue (Sigma-Aldrich, St. Louis, MO, USA) and counted individually. Hemin (referred as heme, Sigma-Aldrich) was prepared by dissolving powder in 0.1 N NaOH and then titrated with 0.1 N HCl to biological pH 7.4, followed by adjustment to the final concentration (10 mM) with saline [89]. Heme stock was then aliquoted and frozen at −80 °C until use; each aliquot was thawed only once. Heme utilizing experiments were carried out in the dark at various concentration of 1–50 μM.

1.2. Transfection and Stable Cell Lines

The following plasmids were used: Flag-HO-1 (full length HO-1), Flag-truncated-HO-1 (C-terminal 23 amino acid truncated, tHO-1), Flag-H25A-HO-1 (enzymatically inactive, H25AHO-1) [15,53,55,56]. 1–2 μg of each plasmid was added to the master mix of Amaxa Kit V in which 1×10^6 PC3 cells were resuspended and Amaxa's protocol was followed for transfection of PC3 cells [53]. 48 h after transfection cells were treated with heme or used for soft agar assays. Of note, the overexpression typically lasts 5–6 days allowing for detecting initial effect on colony growth in soft agar colony culture (2–3 weeks).

1.3. Cell Fractionation

Fractions were isolated using the nuclear/cytoplasmic fractionation kit (BioVision, Milpitas, CA, USA) according to manufacturer's protocol as previously described [19,90].

1.4. Western Blot

Proteins were harvested in lysis buffer (25 mM Tris-HCl, 150 mM NaCl, 1% NP-40, 100 mM NaF, 1 Complete Mini Protease Inhibitor Cocktail Tablet (Roche)). After sonication, lysates were centrifuged at 12,000× *g* at 4 °C for 10 min. Protein concentrations were measured using the BCA Protein Kit (Pierce). 15–35 μg proteins were applied on 4–12% NuPAGE Bis-Tris SDS polyacrylamide gel electrophoresis in MES SDS running system (Novex by Life Technologies) followed by transfer to PVDF membrane (Amersham and Biorad). Following transfer, membranes were blocked in 5% nonfat milk for one hour. The following antibodies were applied rotating overnight at 4 °C: HO-1 (Enzo Laboratories), P-(Ser139)-H2AX (γH2AX) (Cell Signaling), β-Actin (Sigma Aldrich) was used for total lysates while lamin A/C and GAPDH (Cell Signaling Technologies) were used for nuclear and cytoplasmic loading controls, respectively. The following day, after brief washing with Tris-buffered saline, membranes were incubated with HRP conjugated secondary antibodies (Cell Signaling Technologies), followed by chemiluminescent (ECL, Thermo Fisher) detection on film (Bioexpress).

1.5. Immunofluorescence Staining of Cells

Cells were grown on coverslips and fixed with 2% paraformaldehyde for 10 min following washing with PBS 3 times and permeabilization with 0.05% Triton X-100 in PBS for 5 min and then washed twice for 5 min in 1× PBS. Horse serum (7%) diluted in PBS was used for blocking for 30 min. Primary antibodies applied on each section: HO-1 1:200

(Enzo Life Science). Sections were incubated with primary antibodies over night at +4 °C. The following day the slides were placed in PBS twice for 5 min and incubated at RT in darkness for 1 h with secondary antibodies diluted 1:300 (anti-mouse Alexa Fluor® 594, Invitrogen/Life Technologies, Molecular Probes) followed by staining with Hoechst. Slides were carefully washed with PBS and air-dried followed by covering with Gelvatol, Sigma. A Zeiss Apotome Axiovert Fluorescence Microscope was used to evaluate the fluorescence staining.

1.6. Immunoprecipitation

Cells were harvested, homogenized and pulled down experiments with an antibody against HO-1 were performed as described previously [90].

1.7. Helicase Activity

Helicase activity in the lysates or cellular fractions of PC3 cells treated with heme was measured as previously described [89]. Briefly, 1 μg sonicated genomic DNA was incubated with 1 mM ATP, 1× Sally Green, and 10 μg of protein lysates or 1 μg of recombinant HO-1 protein (Enzo Life Sciences) or pull down with antibody against HO-1 in the helicase buffer (200 mM Tris HCl pH 7.6, 25 mM $MgCl_2$, 20 mM DTT, 125 mM KCl, 10% glycerol, 0.5 mg/ml BSA). Reaction was incubated at 37 °C for 30 min and fluorescence was measured at 492 and 530 nm. Percent of unwound substrate was calculated as described in [89].

1.8. Statistics

All data are presented as mean ± standard deviation unless otherwise indicated. Statistical analysis was performed using one-way analysis of variance (ANOVA) followed by the post-hoc Tukey test or using unpaired *T* student test using Graphpad Prism and Excel software. Differences between groups were rated significant at values of $p < 0.05$.
